# Selective Catalytic Oxidation of Cyclohexene with Molecular Oxygen: Radical Versus Nonradical Pathways

**DOI:** 10.1002/cctc.201701538

**Published:** 2018-01-26

**Authors:** Ilse M. Denekamp, Martijn Antens, Thierry K. Slot, Gadi Rothenberg

**Affiliations:** ^1^ Van't Hoff Institute for Molecular Sciences University of Amsterdam Science Park 904 Amsterdam 1098 XH The Netherlands

**Keywords:** autoxidation, doping, heterogeneous catalysis, nanoparticles, sustainable chemistry

## Abstract

We study the allylic oxidation of cyclohexene with O_2_ under mild conditions in the presence of transition‐metal catalysts. The catalysts comprise nanometric metal oxide particles supported on porous N‐doped carbons (M/N:C, M=V, Cr, Fe, Co, Ni, Cu, Nb, Mo, W). Most of these metal oxides give only moderate conversions, and the majority of the products are over‐oxidation products. Co/N:C and Cu/N:C, however, give 70–80 % conversion and 40–50 % selectivity to the ketone product, cyclohexene‐2‐one. Control experiments in which we used free‐radical scavengers show that the oxidation follows the expected free‐radical pathway in almost all cases. Surprisingly, the catalytic cycle in the presence of Cu/N:C does not involve free‐radical species in solution. Optimisation of this catalyst gives >85 % conversion with >60 % selectivity to the allylic ketone at 70 °C and 10 bar O_2_. We used SEM, X‐ray photoelectron spectroscopy and XRD to show that the active particles have a cupric oxide/cuprous oxide core–shell structure, giving a high turnover frequency of approximately 1500 h^−1^. We attribute the high performance of this Cu/N:C catalyst to a facile surface reaction between adsorbed cyclohexenyl hydroperoxide molecules and activated oxygen species.

## Introduction

The allylic oxidation of alkenes is an important chemical reaction. It allows us to keep the double bond and at the same time create a new alcohol or carbonyl function.^1^, ^2^ As such, it is useful across the board, from bulk chemicals and agrochemicals^3^, ^4^ all the way to fine chemicals and fragrances.^5^, ^6^, ^7^ In theory, allylic oxidation is a straightforward exothermic reaction. It requires only O_2_, a free, eco‐friendly and widely available reagent. However, there is a trade‐off: O_2_ has a high activation barrier because of its resonance stabilisation.^8^ Once this barrier is overcome, the active oxygen species often react with hydrocarbons via free‐radical intermediates. These wreak havoc in solution and cause side reactions that all‐too‐often lead to unwanted over‐oxidation products.^9^, ^10^, ^11^

Recently, we showed that this problem can be solved for the specific case of the oxidation of activated alcohols to aldehydes and ketones by using a bifunctional catalyst.^12^ Yet this oxidation was “only” a dehydrogenation reaction. It involved the transfer of protons and electrons without the addition of a new O atom to the substrate. Allylic oxidation is tricky because it is an oxygenation that involves the cleavage of at least one C−H bond and the creation of new C−O or C=O bonds. This requires a direct interaction between the substrate and an active oxygen species, which must then be stopped at the allylic alcohol/ketone stage before “burning” further to carboxylic acids, CO and CO_2_.
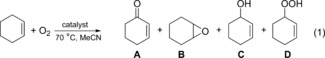



Here we examine the catalytic oxidation of cyclohexene with O_2_ under mild conditions [Eq. 1]. Cyclohexene is a good model compound for two reasons: first, it is a small and symmetric molecule, similar to many starting compounds in chemical synthesis. Second, it is itself industrially important and participates in the synthesis cycles of key C_6_ chemicals such as adipic acid and caprolactone.^13^, ^14^ Building on our preliminary communication on alcohol oxidation,^12^ we designed a set of metal oxide catalysts supported on N‐doped carbons. We used no noble metals and we focussed on abundant transition metal oxides as catalysts.

There are several reports on the allylic oxidation of cyclohexene catalysed by abundant transition metals. In general, the use of O_2_ as an oxidant requires additional activation, either by the addition of 5–10 % of a free‐radical initiator such as H_2_O_2_ or by using elevated temperatures. Zhang and Tang^15^ reported a Cu catalyst on expanded graphite that gave 99 % conversion and 65 % selectivity to 2‐cyclohexene‐1‐one. Yin et al.^16^ and Rossi et al.^17^ used Co‐based catalysts to obtain 94 % conversion and 44 % selectivity, and 90 % conversion and 61 % selectivity, respectively. Peng et al.^18^ used metal‐free N‐doped carbon nanotubes as catalysts. They tested 22 organic solvents and found that acetonitrile gave the best results of 60 % conversion and 39 % selectivity to the 2‐cyclohexene‐1‐one.

Our initial hypothesis was that the allylic oxidation reaction would follow a pathway similar to alcohol oxidation with oxygen activation at the support surface followed by a reaction at the oxide particle. Based on previous reports, we expected the reaction to involve free‐radical intermediates.^10^, ^17^, ^19^ Surprisingly, we found that at least in one case, namely, if we used copper oxide particles supported on N‐doped carbon, there are no free radicals in the solution. In this study, we try to resolve the different pathways that lead to allylic oxidation, with the goal to gain a better understanding of this important reaction.

## Results and Discussion

### Catalyst synthesis and testing

We began by preparing and testing a set of nine d‐block metal oxides supported on the same batch of hierarchically porous N‐doped carbons^20^ (1.2 mmol g^−1^ M/N:C, M=V, Cr, Fe, Co, Ni, Cu, Nb, Mo, W). The catalysts were prepared using vacuum pore impregnation (see Experimental Section for details). To this set of catalysts, we added two blanks: a clean N:C support and a carbon prepared from a citric acid precursor (denoted C_cit_), which has a similar surface area to N:C (≈1500 m^2^ g^−1^) but contains no N. All catalysts were then tested in cyclohexene oxidation by using an autoclave under 10 bar O_2_ and 55 bar Ar, within safe explosion limits. Typically, each autoclave was charged with approximately 25 mmol of cyclohexene, 10 mg of catalyst (a nominal substrate/metal oxide ratio of 2000:1) and 15 mL of MeCN as solvent. Reactions were stirred for 16 h at 1000 rpm and analysed by GC.

Cyclohexene is oxidised to four main products (Table 1): 2‐cyclohexene‐1‐one (**A**), cyclohexene oxide (**B**), 2‐cyclohexene‐1‐ol (**C**) and 2‐cyclohexene‐1‐hydroperoxide (**D**; herein the ketone, epoxide, alcohol and hydroperoxide, respectively). The rest of the products were over‐oxidation products, mainly CO and CO_2_. Products **A**–**C** were determined directly by GC. The hydroperoxide **D** could not be observed by GC and was quantified by reacting each sample with PPh_3_ (see Experimental Section for details). Control experiments confirmed that the internal standard, cyclohexane, showed no conversion under these reaction conditions. Further, in the absence of any catalyst, the background reaction at 70 °C gives only 22 % conversion, most of it to CO and CO_2_ (Table 1, entries 1–3). The addition of porous carbon does not change the conversion but reduces the amount of over‐oxidation slightly, possibly because of radical‐scavenging by the carbon surface sites.^21^ In the presence of pristine N:C, the conversion more than doubles to approximately 50 %. Moreover, the selectivity to the ketone **A** increases to 28 %, at the expense of the hydroperoxide **D**. Indeed, we showed recently that these porous N:C materials are excellent oxygen reduction catalysts,^20^ yet these results also point to a N:C‐catalysed route from **D** to **A** (vide infra).

**Table 1:** Cyclohexene oxidation in the presence of different catalysts.^[a]^ 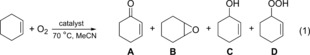

Entry	Catalyst	Conversion [%]	Selectivity [%]				
			**A**	**B**	**C**	**D**	Other
1	None	22	8	2	0	10	80
2	C_cit_	23	14	2	0	17	67
3	N:C	49	28	3	0	5	64
4	W@N:C	43	17	4	0	12	67
5	Ni@N:C	44	17	3	0	17	63
6	Mo@N:C	45	17	5	0	11	69
7	Fe@N:C	53	23	3	0	3	74
8	Nb@N:C	58	25	6	0	8	61
9	V@N:C	64	20	15	0	2	63
10	Cr@N:C	66	32	7	1	4	56
11	Cu@N:C	71	47	9	16	4	24
12	Co@N:C	80	38	6	6	6	44

[a] Reaction conditions: 10 bar O_2_; 2,5 mL (24,7 mmol) cyclohexene; 0.5 mL (1.85 mmol) cyclohexane (IS); 10 mg catalyst; 15 mL MeCN; stirred in an autoclave (1000 rpm); 70 °C; 16 h.

The addition of W, Ni, Mo, Fe or Nb does not change the results significantly (Table 1, entries 4–8). For some of these catalysts, the selectivity to **A** is lower than that of the pristine N:C support, which may reflect the blocking of labile sites on the support by metal oxide particles. However, the catalysts that contain V, Cr, Cu and Co showed a significant increase in conversion (entries 9–12). Vanadium oxide (V/N:C), which is known as a good epoxidation catalyst,^22^, ^23^ gives a high selectivity to the epoxide **B**. The remaining three catalysts are interesting: they are the only ones to give measureable yields of the alcohol **C**. All three give less hydroperoxide compared with the blanks, which indicates a pathway from **D** to **C**. Cobalt oxide gives the highest conversion. However, copper oxide gives the highest selectivity to the ketone **A** with a remarkably low amount of over‐oxidation products. Even at this un‐optimised stage, the Cu/N:C catalyst gives a combined ketone+alcohol yield of nearly 45 % with a minimum turnover number (TON) >1400 and turnover frequency (TOF) >88 h^−1^ (the actual TON and TOF per site are much higher because most of the copper oxide is not accessible, see discussion below). Therefore, we focussed our investigation on these two catalysts.

Control reactions in which we used equivalent amounts of cobalt oxide supported on γ‐alumina showed lower conversions and more side‐products, which confirms the importance of the N:C support (Table 2, entry 2). Copper oxide supported on γ‐alumina shows a good conversion but with a lower selectivity and more side‐products than that supported on N:C, making the γ‐alumina‐supported catalyst less favourable (entry 6). Notably, the difference in the surface area between the carbon and the γ‐alumina was corrected for by increasing the catalyst amount accordingly. To boost the number of free radicals at the start of the reaction, we added H_2_O_2_ (13 mol % relative to the substrate, entry 3).^5^, ^7^ H_2_O_2_ can decompose into water and oxygen under these reaction conditions. The water molecules themselves do not change the conversion and selectivity (the H_2_O_2_ solution is already 90 % water), but the decomposition of H_2_O_2_ affects the reaction by releasing free radicals into the solution. The addition of H_2_O_2_ increased the conversion but did not change the selectivity significantly. A similar increase was observed if the reaction was performed at 80 °C. With copper oxide, however, the addition of H_2_O_2_ or an increase of the reaction temperature affected both the conversion and the selectivity (entries 7 and 8). The conversion increased to 85 %, and the combined selectivity to **A**+**C** increased to 70 %. Importantly, this increase in selectivity came at the expense of the over‐oxidation products (unlike with Co, with which there was still a lot of over‐oxidation products). To our minds, this was counter‐intuitive: we would assume that the addition of an initiator such as H_2_O_2_ or an increase of the temperature would lead to more CO and CO_2_. These results led us to think that perhaps the copper oxide catalysed reaction is not a simple free‐radical process. Previous reports in which the oxidation kinetics of cyclohexene and [D_10_]cyclohexene are compared show a clear primary isotope effect (*k*_H_/*k*_D_=8.2), which indicates that the rate‐determining step involves C−H bond scission.^10^ Moreover, the addition of a radical scavenger quenched the reaction.^10^, ^15^ To check if this also applies our system, we ran additional control experiments in the presence of 6 mol % of 2,6‐di‐*tert*‐butyl‐4‐methylphenol (BHT; see details in the Experimental Section). The addition of BHT to the reaction mixture that contained the N:C support or the Co/N:C catalyst stopped the reaction completely (Table 3, cf. entries 2 and 4 with 1 and 3). However, if we added BHT to the Cu/N:C‐catalysed reaction, there was only a slight decrease in the conversion and selectivity (from 85 to 76 % and from 53 to 46 %, respectively; entries 5 and 6). This shows that although the reactions catalysed by metal‐free N:C and by Co/N:C are definitely free‐radical processes, the Cu/N:C‐catalysed reaction is not affected by free‐radical scavengers (these experiments were repeated multiple times by different people to ensure their repeatability and reproducibility). Therefore, we conclude that in the Cu‐catalysed system, there are no free radicals in solution.

**Table 2:** Oxidation of cyclohexene with various copper oxide and cobalt oxide catalysts.^[a]^

Entry	Catalyst	*T* [°C]	Conversion [%]	Selectivity [%]				
				**A**	**B**	**C**	**D**	Other
1	Co@N:C	70	80	38	6	6	6	44
2	Co@Alu	70	56	20	3	0	3	74
3^b^	Co@N:C	70	87	43	6	11	nd^[c]^	40
4	Co@N:C	80	87	41	12	5	nd^[c]^	42
5	Cu@N:C	70	71	47	9	16	4	24
6	Cu@Alu	70	77	31	7	2	0	60
7^b^	Cu@N:C	70	86	61	15	8	nd^[c]^	16
8	Cu@N:C	80	85	53	10	17	nd^[c]^	20

[a] Reaction conditions: 10 bar O_2_; 2.5 mL (24.7 mmol) cyclohexene; 0.5 mL (1.85 mmol) cyclohexane (IS); 10 mg carbon catalyst, 73 mg alumina catalyst; 15 mL MeCN; stirred in an autoclave (1000 rpm); 16 h. [b] 1.0 mL H_2_O_2_ added (10 wt %, 3.3 mmol, 13 mol % based on cyclohexene). [c] Not determined.

**Table 3:** Effects of the addition of free‐radical scavengers.^[a]^

Entry	Catalyst	Addition	Conversion [%]	Selectivity [%]			
				**A**	**B**	**C**	Other
1^[b]^	N:C	–	49	28	3	0	69
2^[b,c]^	N:C	BHT	0	0	0	0	0
3	Co@N:C	–	87	41	12	5	42
4^[c]^	Co@N:C	BHT	0	0	0	0	0
5	Cu@N:C	–	85	53	10	17	20
6^[c]^	Cu@N:C	BHT	76	46	10	20	24

[a] Reaction conditions: 10 bar O_2_; 2.5 mL (24.7 mmol) cyclohexene; 0.5 mL (1.85 mmol) cyclohexane (IS); 10 mg carbon catalyst; 15 mL MeCN; stirred in an autoclave (1000 rpm); 80 °C; 16 h. [b] Reaction temperature 70 °C. [c] 354 mg BHT added (7 mol % based on cyclohexene).

**Figure 1:**
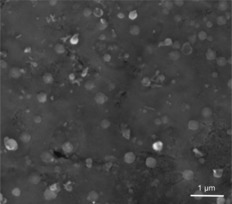
Scanning electron micrograph of Cu/N:C at ×15 000 magnification (an image with particle measurements is included in the Supporting Information).

SEM images (Figure 1) of the Cu/N:C catalyst showed spherical copper oxide particles of approximately 200–250 nm in diameter distributed evenly across the surface. Unlike the support, the particles are non‐porous. Consequently, most of the copper oxide is “inside” the particle and unavailable for catalysis. If we consider that the active outer shell is approximately five atomic layers (≈2 nm in thickness), the actual active catalyst comprises only 0.1 wt %. Accordingly, the actual TON of this catalyst would be >24 000 with a corresponding TOF of >1500 h^−1^.

**Figure 2:**
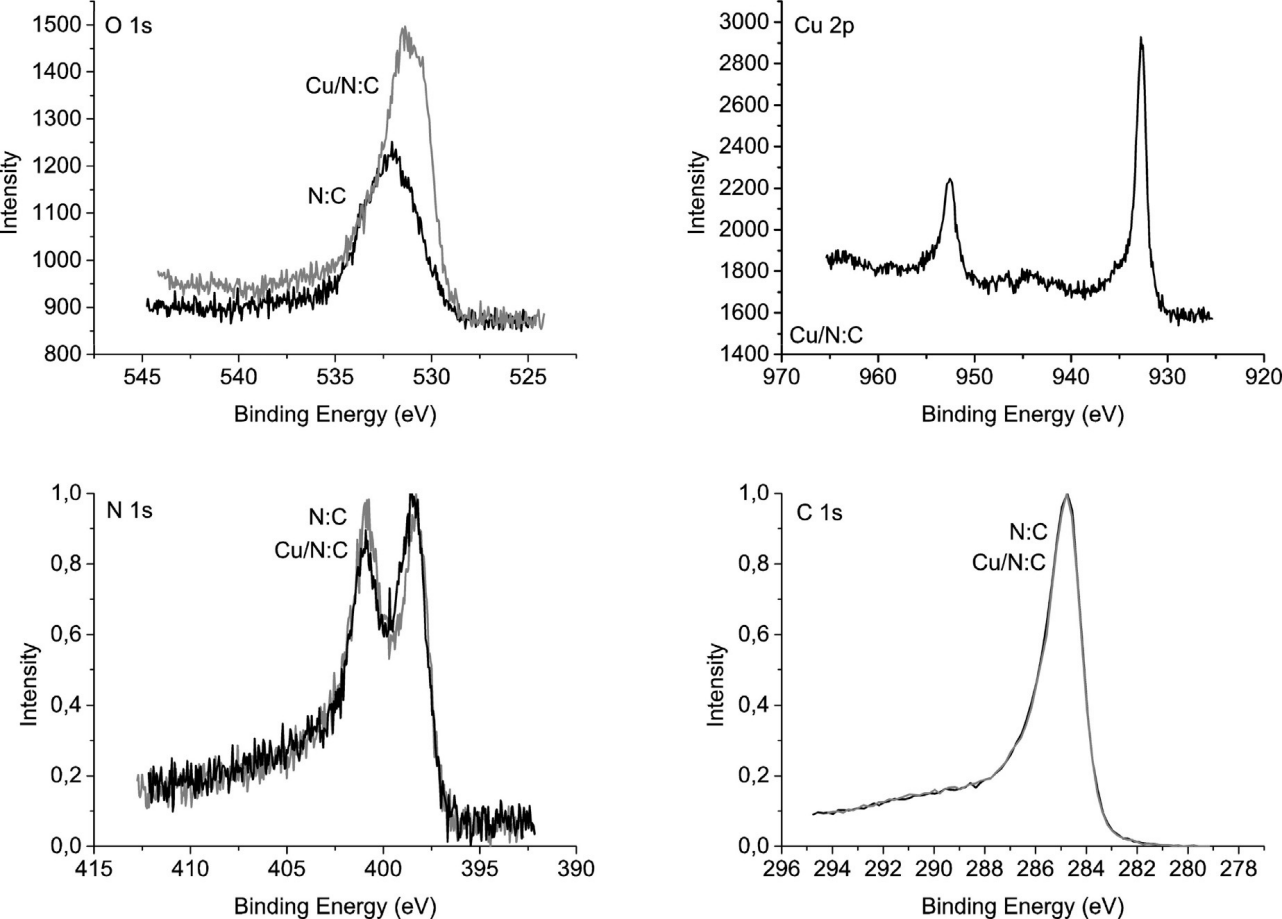
XPS spectra of Cu/N:C and pristine N:C that show the O 1s, Cu 1/2p and 3/2p, N 1s and C 1s binding energies. Impregnation of copper oxide on the N:C increases the O content but does not affect the N or C peaks. Notably, the N and C spectra are normalised for clarity.

We used X‐ray photoelectron spectroscopy (XPS; Figure 2) to show that the impregnation of the N:C surface with copper oxide does not affect the N binding energy. This suggests that the copper oxide is not coordinated to surface N atoms. The impregnation increases the intensity of the O 1s peak, which indicates a higher oxygen content in the sample. For Cu, the XPS spectrum shows the typical Cu 2p_1/2_ and Cu 2p_3/2_ peaks, which can be assigned to both Cu^+^ and Cu^2+^. Yet the characteristic CuO peak at a binding energy of 945 eV is absent, which supports the presence of Cu_2_O^24^ (metallic Cu is unlikely at such low treatment temperatures^25^ and if we consider the increase in the O signal). The carbon peak is not affected by impregnation with Cu. However, powder XRD patterns of the catalyst show CuO as the major component in the particles (see details in the Supporting Information). These results are consistent with a CuO–CuO_2_ core–shell structure as XRD measures the entire particle, whereas XPS penetrates only a few atomic layers.^26^ Therefore, we suggest that during the thermal treatment following the impregnation step, the adsorbed Cu(NO_3_)_2_ precursor is first converted to CuO and NO_2(g)_. As the temperature approaches 300 °C, the Cu_2_O shell starts to form. Indeed, temperature‐programmed reduction measurements indicate the presence of multiple copper oxides (details in Supporting Information). Similarly, thermogravimetric analysis of the pristine N:C and the Cu/N:C samples shows that the latter decomposes at a lower temperature (400 vs. 500 °C, respectively). This supports the hypothesis that Cu partially oxidises the surface to create more labile sites (see details in the Supporting Information).

### Mechanistic considerations

From these results, we propose two alternatives for the catalytic allylic oxidation of cyclohexene with O_2_. The first follows the traditional free‐radical route and pertains to the Co, Fe, Cr, Mo, V, Ni, Nb and W/N:C catalysts. Here, O_2_ is either activated thermally or in a redox process on the N:C surface. The insertion of this activated oxygen into the allylic C−H bond gives the cyclohexenyl hydroperoxide **D**. This can then either rearrange to give the ketone **A** or undergo scission to give oxo and peroxo radicals that propagate a chain oxidation reaction.^2^, ^15^ Accordingly, this pathway, which involves free radicals in the bulk solution, is quenched readily if BHT is added.

**Figure 3:**
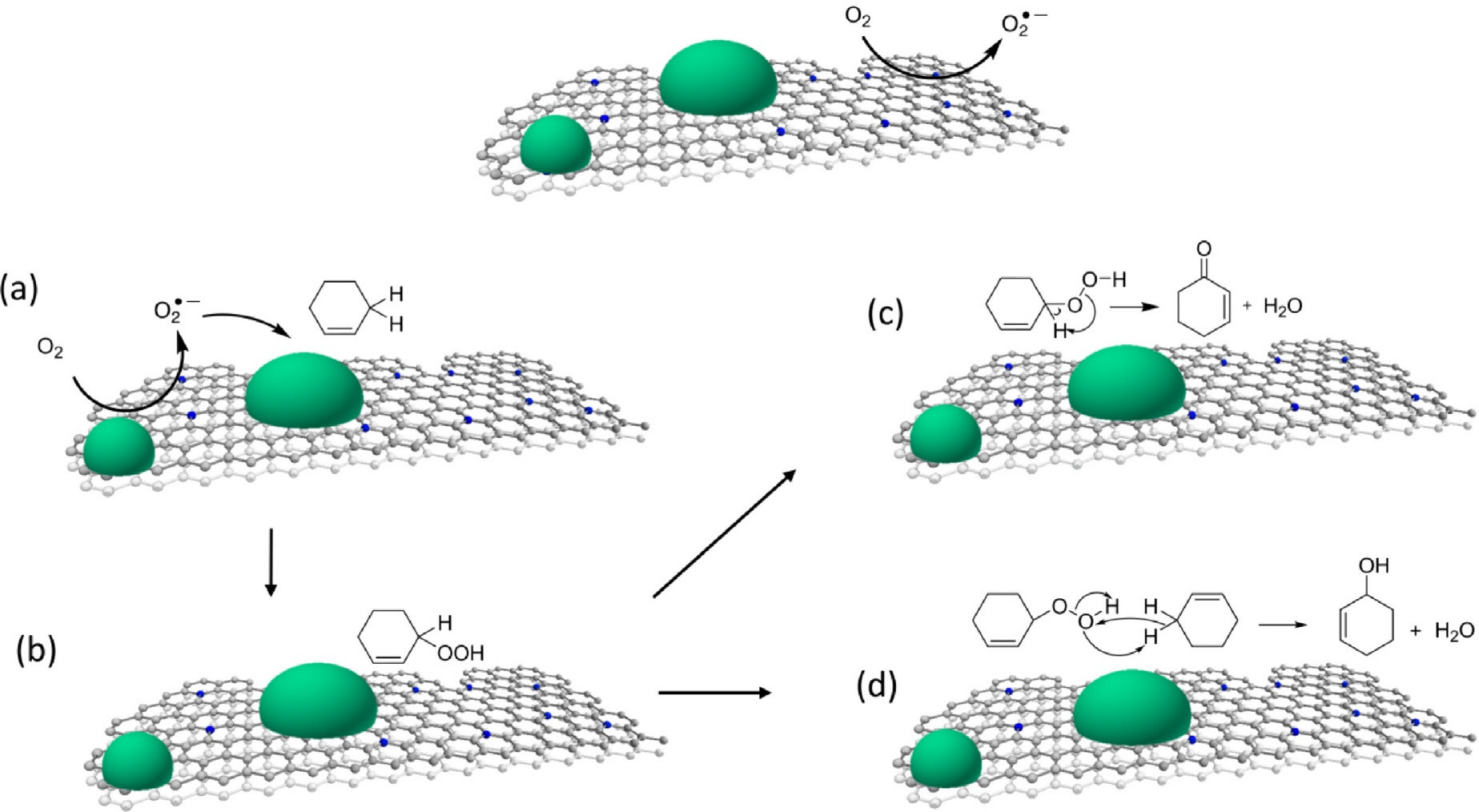
Proposed reaction pathways for the catalytic oxidation of cyclohexene with O_2_ in the presence of Cu/N:C. a) Oxygen activation at the support surface followed by radical migration into solution. b) Insertion of activated oxygen into the allylic C−H bond to give the adsorbed hydroperoxide **D** followed by either c) rearrangement to the ketone **A** and water or d) reaction with another cyclohexene molecule to give two molecules of the alcohol **C**.

Conversely, in the presence of Cu/N:C, there are no free radicals in the bulk solution. Oxygen can still be activated at the N:C surface sites but now there are two options: the small amounts of short‐lived activated oxygen species (e.g., O_2_^−.^ radical anions) that travel into solution will be quenched by BHT (Figure 3 a, cf. also the difference in conversion between entries 5 and 6 in Table 3). The BHT molecules are too bulky to enter the micropores. Therefore, they will quench only the radicals in the solution. Conversely, the activated oxygen species that are close enough to diffuse to a supported copper oxide particle^27^ can react there with cyclohexene to form an adsorbed hydroperoxide (Figure 3 b). This adsorbed hydroperoxide can undergo two reactions. The first is rearrangement and dehydration to give the ketone **A** and a molecule of water (Figure 3 c).^9^, ^28^ The second is a disproportionation reaction with another cyclohexene molecule to give two molecules of cyclohexene‐1‐ol **C** (Figure 3 d). Compared with the other metal oxides, the scission of the RO−OH bond on the copper oxide surface is apparently much slower. This means that fewer free radicals are released into the solution, which gives enough time for the rearrangement and disproportionation reactions.

Interestingly, there is a marked difference between the oxidation of activated alcohols, which we reported earlier,^12^ and that of cyclohexene. With an activated alcohol substrate such as cinnamyl alcohol, the N:C support is required for oxygen activation. There, no reaction was observed for copper oxide particles supported on C_cit_, an analogous porous carbon with no N dopants. Cyclohexene oxidation, however, does proceed in the presence of Cu/C_cit_, which shows that the allylic oxidation in this case is easier. This is supported by the results of Gray and co‐workers^10^ who showed that the allylic C−H bond scission is rate‐determining and by the fact that this bond is weaker than the alcohol C−H bond (83 and 96 kcal mol^−1^, respectively^29^, ^30^).

In all cases, the epoxide **B** probably forms by another pathway.^4^, ^31^ Cyclohexene molecules can interact with M=O groups on the particle surface to give the epoxide and a labile surface site that is then re‐oxidised by incoming oxygen.^24^

## Conclusions

The catalytic oxidation of cyclohexene with O_2_ can follow different pathways that depend on the type of catalyst. In the presence of transition metal oxide nanoparticles supported on N‐doped carbons, the key step is the insertion of O_2_ into the allylic C−H bond to give the cyclohexenyl hydroperoxide. This reaction can be enhanced by oxygen activation at the N‐doped carbon surface. In most cases, the allylic oxidation follows a free‐radical pathway. However, in the presence of Cu/N:C the reaction does not release free radicals into solution. This enables a more selective reaction at the copper oxide surface, which probably involves cuprous oxide sites.

## Experimental Section

### Materials and instrumentation

GC was performed by using a PerkinElmer Clarus 580 instrument. This system was equipped with a flame ionisation detector and autosampler (G4513A). A 30 m×32 mm I.D. Rxi‐5 ms fused silica crossbond diphenyl dimethyl polysiloxane column with a film thickness of 0.25 μm was used. The injector volume was 1 μL, and the flow was 100 mL min^−1^ of He carrier gas. The temperature program was 40 °C, 20 °C min^−1^, 160 °C for 2 min. SEM was performed by using a Verios‐460 microscope (FEI) at an accelerating voltage of 5 kV with a working distance of 2–5 mm. Powder XRD patterns were obtained by using a MiniFlex II diffractometer by using Ni‐filtered CuK_α_ radiation. The X‐ray tube was operated at 30 kV and 15 mA with a 0.01° step and 1 s dwell time. XPS measurements were performed by using a PHI VersaProbe II scanning XPS microprobe (Physical Instruments AG, Germany) using a monochromatic AlK_α_ X‐ray source with a power of 24.8 W and a beam size of 100 μm. The spherical capacitor analyser was set at a 45° take‐off angle with respect to the sample surface. The pass energy was 46.95 eV to yield a full width at half maximum of 0.91 eV for the Ag 3d_5/2_ peak. Peaks were calibrated using the C 1s position. Curve fitting was performed using the XPSPeak 4.1 software package. All chemicals were obtained from commercial sources (>99 % pure) and were used as received. Temperature‐programmed reduction (TPR) was performed by placing 25 mg of sample sandwiched between two quartz wool plugs in a quartz tube reactor (4 mm i.d.). After purging with N_2_, a flow of 5 % H_2_ in N_2_ was applied. The system was allowed to equilibrate and then heated at 5 °Cmin^−1^ to 800 °C (no hold time).

### Preparation of the N‐doped carbon support

The N:C support samples were prepared following the procedure published by Eisenberg et al.^20^ Briefly, nitrilotriacetic acid (NTA) was mixed in a 1:1 ratio with magnesium carbonate. This was dissolved in de‐ionised water, stirred for 10 min at 85 °C, and cooled to RT. The solid was then precipitated by adding an excess of ethanol and chilling in an ice bath for 2 h. The white solid was scraped out, dried at 40 °C for 48 h, and ground into a fine white powder. This powder was then pyrolysed in Ar at 900 °C. The MgO particles were washed with 3×500 mL of 0.5 m citric acid. The resulting crude N:C sample was dried at 120 °C for 2 h and treated under Ar at 1000 °C for 1 h.

### Preparation of M/N:C catalysts

This is a modification of the procedure published by Slot et al.^12^ The N‐doped carbon support (100 mg) was placed in a small vial with a septum. The air was removed carefully by using a needle, and an aqueous solution of the desired metal precursor salt (0.2 mL, which corresponds to a nominal loading of 1 mmol m^−2^) was added to the vial under continuous stirring. The vial was shaken vigorously for 2–3 min to create a uniform solid paste, which was then dried at 85 °C for 12 h. Each catalyst was then heat‐treated at 300 °C under Ar (except for Nb/N:C, which was treated at 700 °C) and cooled to RT. The different M/N:C catalysts were prepared from their respective precursors salts: Co(NO_3_)_2_⋅6 H_2_O, Cu(NO_3_)_2_⋅3 H_2_O, Fe(NO_3_)_3_⋅9 H_2_O, NH_4_VO_3_ (dissolved using 2 equiv. of oxalic acid), Cr(NO_3_)_3_⋅9 H_2_O, (NH_4_)_6_Mo_7_O_24_⋅4 H_2_O, Ni(NO_3_)_2_⋅6 H_2_O, C_10_H_5_NbO_20_⋅*x* H_2_O and (NH_4_)_10_W_12_O_41_⋅5 H_2_O. The Co/alumina catalyst sample was prepared similarly from γ‐Al_2_O_3_ (Ketjen; ground and sieved to 200–400 nm) and Co(NO_3_)_2_⋅6 H_2_O stock solution (0.8 mL, 0.55 m).

### Catalytic oxidation of cyclohexene

This is a modification of the procedure published by Cao et al.^32^ A 75 mL autoclave lined with a 50 mL Teflon insert was loaded with cyclohexene (2.5 mL, 24.7 mmol), cyclohexane (0.5 mL, internal standard), acetonitrile (solvent, 15 mL), catalyst (10 mg M/N:C carbon or 73 mg Co/alumina) and a stirring bar (30 mm). The autoclave was sealed, flushed with Ar and O_2_ twice before the final O_2_ (10 bar) and Ar (55 bar) atmosphere was applied. The autoclave was then heated to 70 °C for 16 h with stirring at 1000 rpm. After 16 h, the autoclave was cooled to RT. Acetone (5 mL) was added to the sample, and the reaction mixture was filtered using 0.45 μm PTFE syringe filters and analysed by using GC.

The presence of free radicals in solution was tested by adding BHT (354 mg, 6 mol %) to the reaction at *t*=0 and then following the above procedure. Reactions were performed in triplicate, and all GC analyses were performed in duplicate.

### Quantification of the cyclohexenyl hydroperoxide D

The hydroperoxide **D** cannot be measured directly by using GC because of its instability. Instead, we quantified it by comparing a control reaction sample to one in which triphenylphosphine (PPh_3_, 30 mg, 1 mol %) was added. The sample was shaken for 1 min, and heat was generated as the PPh_3_ reacts with the hydroperoxide **D** to give PPh_3_O and the alcohol **C** [Eq. 2]. After this reaction, the sample was analysed by GC and compared to its untreated counterpart. The subtraction of the initial amount of the alcohol formed in the control reaction from the amount of the alcohol after the addition of PPh_3_ gives the amount of hydroperoxide **D** in the original sample.^6^, ^33^

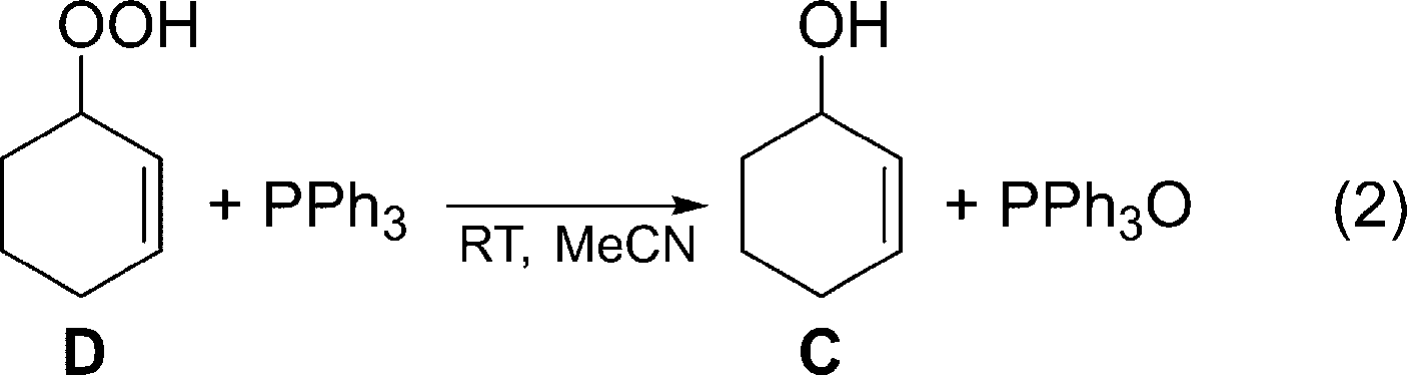



## Conflict of interest

*The authors declare no conflict of interest*.

## Supporting information

As a service to our authors and readers, this journal provides supporting information supplied by the authors. Such materials are peer reviewed and may be re‐organized for online delivery, but are not copy‐edited or typeset. Technical support issues arising from supporting information (other than missing files) should be addressed to the authors.

Supplementary
