# Supplementary material for: Selective Catalytic Oxidation of Cyclohexene with Molecular Oxygen: Radical Versus Nonradical Pathways
Source: ChemCatChem. 2018 Jan 26;10(5):1035–41. doi: 10.1002/cctc.201701538 (PMC5873426; doi:10.1002/cctc.201701538)
Supplement: Supplementary file 1 — Supplementary [file CCTC-10-1035-s001.pdf]

Heterogeneous & Homogeneous & Bio- & Nano-
CHEMCATCHEM
CATALYSIS

Supporting Information

Selective Catalytic Oxidation of Cyclohexene with Molecular Oxygen: Radical Versus Nonradical Pathways

Ilse M. Denekamp, Martijn Antens, Thierry K. Slot, and Gadi Rothenberg*[a]

cctc_201701538_sm_miscellaneous_information.pdf

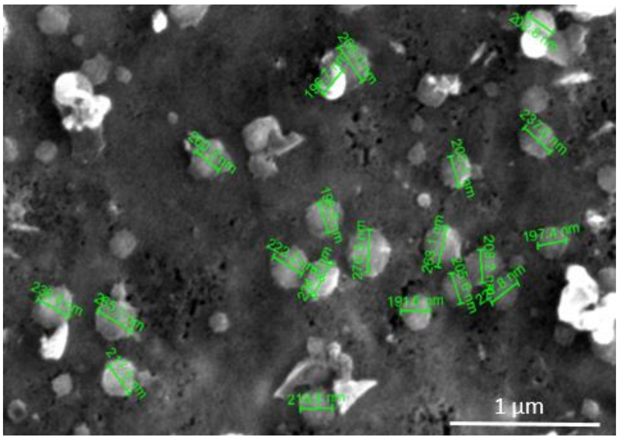

Figure S1. Scanning electron micrographs of Cu/N:C. The size of the copper oxide is ca. 200-250 nm

Scanning electron microscopy (SEM) of Cu/N:C catalyst with particle measurements. The average size of the copper oxide spherical particles is ca. 200-250 nm. Furthermore, the clusters are evenly distributed onto the support surface.

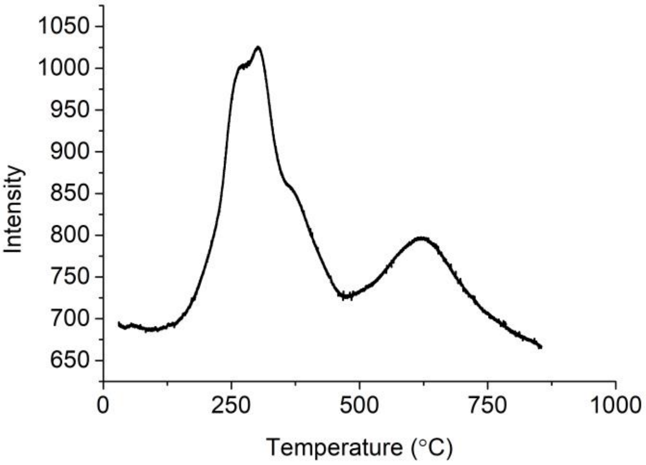

Figure S2. Temperature programmed reduction (TPR) spectrum for Cu/N:C.

The temperature programmed reduction (TPR) shows multiple copper oxide species presence inside the copper oxide catalyst. At 300 °C copper oxide is reduced, where multiple peaks indicate multiple copper oxide species. At 600 °C the support N:C is reduced. [1]

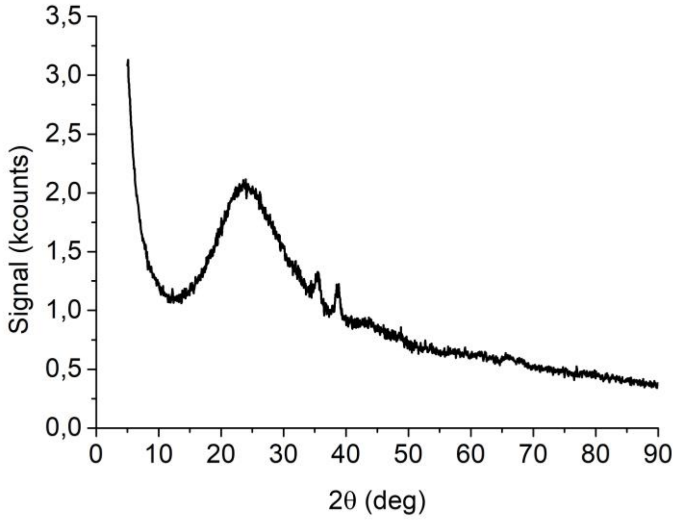

Figure S3. X-ray powder diffraction (XRD) spectrum for Cu/N:C.

X-ray diffraction showed a broad peak at 25° which is typical for carbon. The small peaks at 36.1° (-111) and at 39° (111) are typical for CuO.[2]

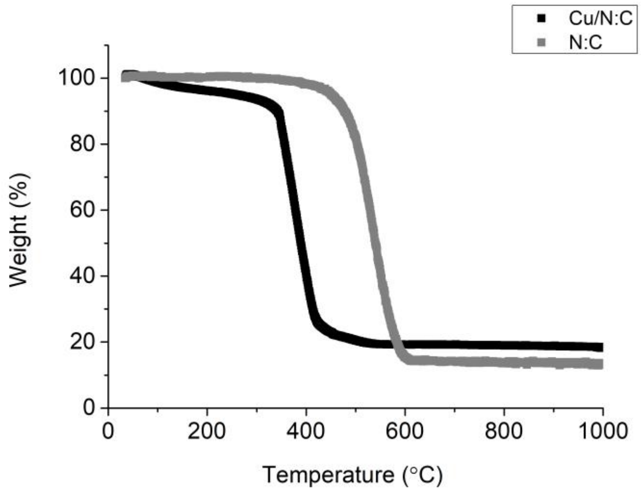

Figure S4. Thermal gravimetric analysis (TGA) curves of Cu/N:C and plain N:C.

TGA showed the mass loss of N:C at 500°C, whereas the Cu/N:C has a mass loss at a lower temperature. This difference indicates that the copper oxidises the nitrogen-doped surface during the treatment, thereby lowering the temperature of the mass loss. The difference in weight percentage at the end of the measurement around 600 °C is the copper oxide that is present in the Cu/N:C sample.

References

[1] T. K. Slot, D. Eisenberg, D. van Noordenne, P. Jungbacker, G. Rothenberg, *Chem. Eur. J.* **2016**, *22*, 12307–12311.

[2] J. Morales, L. Sánchez, F. Martín, J. R. Ramos-Barrado, M. Sánchez, *Electrochim. Acta* **2004**, *49*, 4589–4597.
